# Cuproptosis-related gene *DLAT* serves as a prognostic biomarker for immunotherapy in clear cell renal cell carcinoma: multi-database and experimental verification

**DOI:** 10.18632/aging.205181

**Published:** 2023-11-07

**Authors:** Shuaishuai Huang, Congbo Cai, Kena Zhou, Xue Wang, Xue Wang, Dong Cen, Guobin Weng

**Affiliations:** 1 Department of Laboratory, Ningbo Urology and Nephrology Hospital, Ningbo, China; 2 Department of Emergency, Ningbo Urology and Nephrology Hospital, Ningbo, China; 3 Shanghai Jiao Tong University School of Medicine, Shanghai, China; 4 Department of Ultrasound, Ningbo Urology and Nephrology Hospital, Ningbo, China

**Keywords:** *DLAT*, clear cell renal cell carcinoma, prognosis, tumor microenvironment, immune response

## Abstract

Objective: Renal clear cell carcinoma (ccRCC) is the most common type of renal cancer. Here we aim to explore the prognosis and immunotherapeutic value of copper death-related gene *Dihydrolipoamide S-acetyltransferase* (*DLAT)* in ccRCC.

Methods: The mRNA and protein expressions and methylation level of *DLAT*, as well as the relation of *DLAT* to survival prognosis, clinical characteristics, biological function, and immune microenvironment and responses in patients with ccRCC were evaluated using multiple databases. In addition, 75 paired ccRCC tissue samples and 3 kinds of cell lines were tested for experimental validation.

Results: Bioinformatics analysis of multiple databases, qRT-PCR, and western blot verified that *DLAT* expression in ccRCC was lower than that in paracancerous tissues. Patients with low expression of *DLAT* had a lower survival rate, worse clinical prognosis, stronger immune cell infiltration and expression of immunosuppressive points, and higher tumor immune dysfunction and exclusion (TIDE) scores.

Conclusions: *DLAT* was identified as an independent prognostic factor in ccRCC and was closely related to the prognosis and immune responses of patients with ccRCC.

## INTRODUCTION

Renal cell carcinoma (RCC) is a common and highly malignant tumor of the urinary system [1]. According to the latest GLOBOCAN estimations, 431,288 people worldwide are newly diagnosed with RCC, accounting for 2.2% of all new cancer cases [2]. RCC is a heterogeneous group of tumors, of which clear cell renal cell carcinoma (ccRCC) accounts for 75–90% [3–5]. The prognosis of early ccRCC is good; however, the 5-year survival rate of patients with metastatic ccRCC is less than 20% [6]. In recent years, targeted therapy and immune checkpoint inhibitors (ICIs) have been shown to be crucial and effective strategies for treating advanced RCC [7, 8]. Therefore, exploring reliable predictors of immunotherapy is critical for delivering precision therapy in ccRCC.

Cuproptosis, a copper-triggered modality of mitochondrial cell death, plays an important role in ccRCC [9]. Protein fatty acylation is a highly conserved post-translational modification of lysine [10]. Ferredoxin 1 (*FDX1*) is an upstream regulator of protein fatty acylation, and the Dihydrolipoamide S-acetyltransferase (*DLAT*) acts as a downstream component of *FDX1* [10–12]. During this process, copper ions directly bind and contribute to the oligomerization of lipoylated *DLAT*, thus regulating the tricarboxylic acid (TCA) cycle [12]. Multiple studies have shown that *DLAT* is associated with the prognosis of liver, gastric, and pancreatic cancer [13–15]. However, its expression level and predictive value for ccRCC remain unclear.

In the present study, we aimed to investigate the role of *DLAT* in ccRCC. Multiple databases and ccRCC cell lines were used to assess the association between *DLAT* and clinical performance of ccRCC patients. Here, *DLAT* methylation, protein transcription, and immunotherapy value were evaluated. Our findings were verified using quantitative real-time PCR (qRT-PCR) and western blot in 75 ccRCC cohorts collected by our institute (Ningbo Urology and Nephrology Hospital [NBUNH]) and three cell lines.

## RESULTS

### Expression analysis of *DLAT*

**Figure 1:**
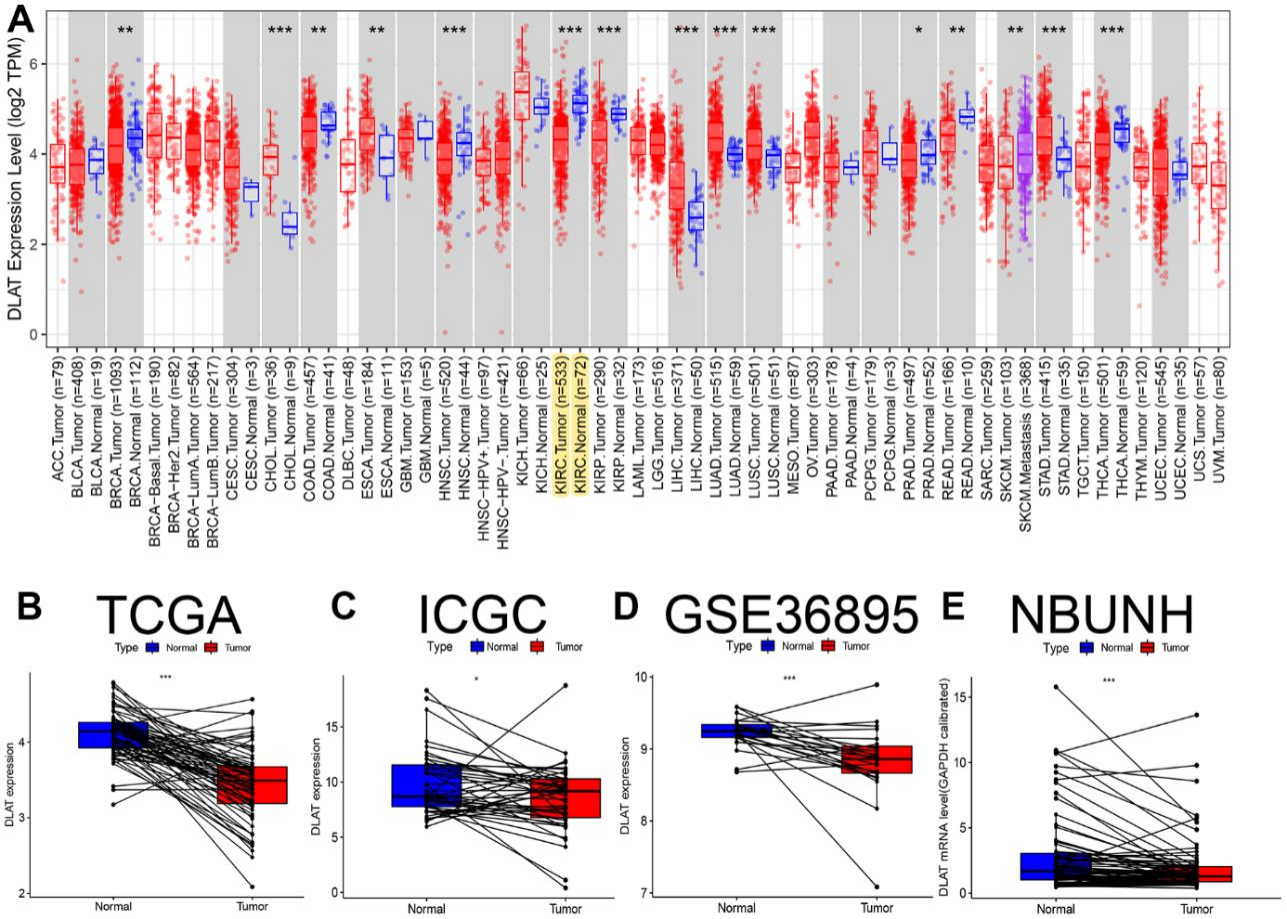
**Pan-cancer analysis and expression level verification of *DLAT* in ccRCC.** (**A**) Expression levels of *DLAT* in multiple tumors. Yellow background color indicates the expression of *DLAT* in KIRC. (**B**) Comparison of paired *DLAT* expression levels in ccRCC from TCGA. (**C**) Comparison of paired *DLAT* expression levels in ccRCC from ICGC (RECA-EU). (**D**) Comparison of paired *DLAT* expression levels in ccRCC from GEO (GSE36895). (**E**) Comparison of paired *DLAT* expression levels in ccRCC from NBUNH data cohort. *: *P*< 0.05; **: *P*< 0.01; ***: *P*< 0.001.

The TCGA RNA-seq analysis showed that mRNA levels of *DLAT* were lower in cancer tissues compared with the paracancerous tissues in BRCA, COAD, HNSC, KIRC, KIRP, PPAD, READ, and THCA (Figure 1A). Moreover, *DLAT* mRNA levels were lower in renal cancer tissues than that in paracancerous tissues from the TCGA-KIRC, ICGC (RECA-EU) and GEO (GSE36895) databases (Figures 1B–1D). Consistent with above database, the results were statistically significant in paired comparisons in our NBUNH cohort (*P*< .05) (Figure 1E).

### Analysis of survival and prognosis of *DLAT* expression

**Figure 2:**
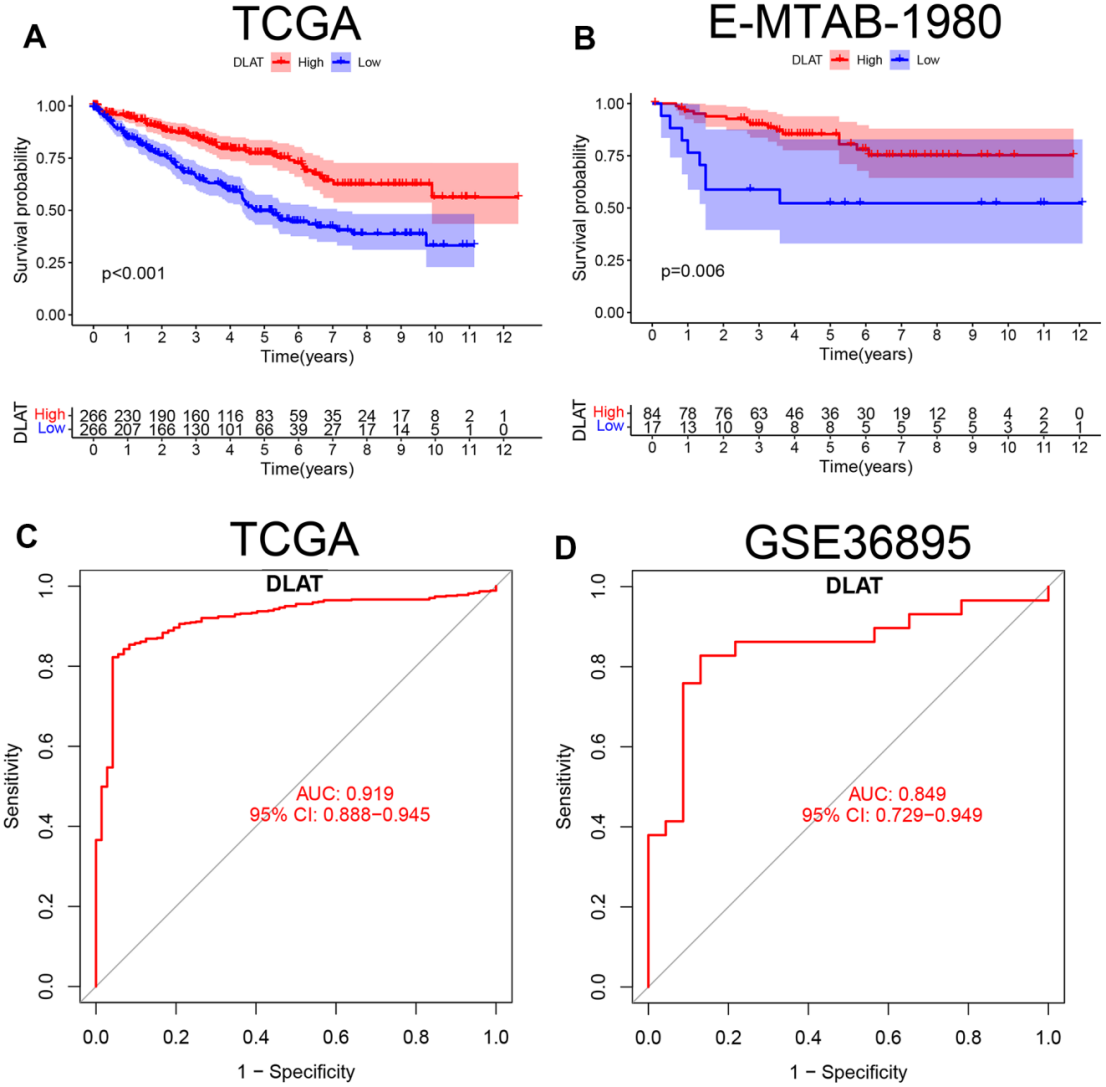
**K-M survival curve and ROC of *DLAT* in ccRCC.** (**A**) Kaplan-Meier survival curve between high- and low-expression of *DLAT* groups from TCGA. (**B**) Kaplan-Meier survival curve between high- and low-expression of *DLAT* groups from E-MTAB-1980. (**C**) ROC curves and AUC values of *DLAT* differences in ccRCC tissue and healthy controls from TCGA. (**D**) ROC curves and AUC values of *DLAT* differences in ccRCC tissue and healthy controls from GSE36859.

According to the median expression levels of *DLAT* in the TCGA cohort, patients were divided into high- and low-expression groups. The Kaplan–Meier curve showed that the overall survival (OS) in the low-expression group was lower than that in the high-expression group (*P*< .01, Figure 2A). In the E-MTAB-1980 cohort, the same results were obtained when the optimal cutoff *DLAT* expression level was selected (*P*< .01, Figure 2B). The ROC curve showed that *DLAT* performed well in distinguishing between benign and malignant tumors. The AUC value in the TCGA database was 0.919 (95% CI=0.888–0.945) (Figure 2C), whereas in the GSE36895 cohort, it was 0.849 (95% CI=0.729–0.949) (Figure 2D).

### Clinical value of *DLAT*

**Figure 3:**
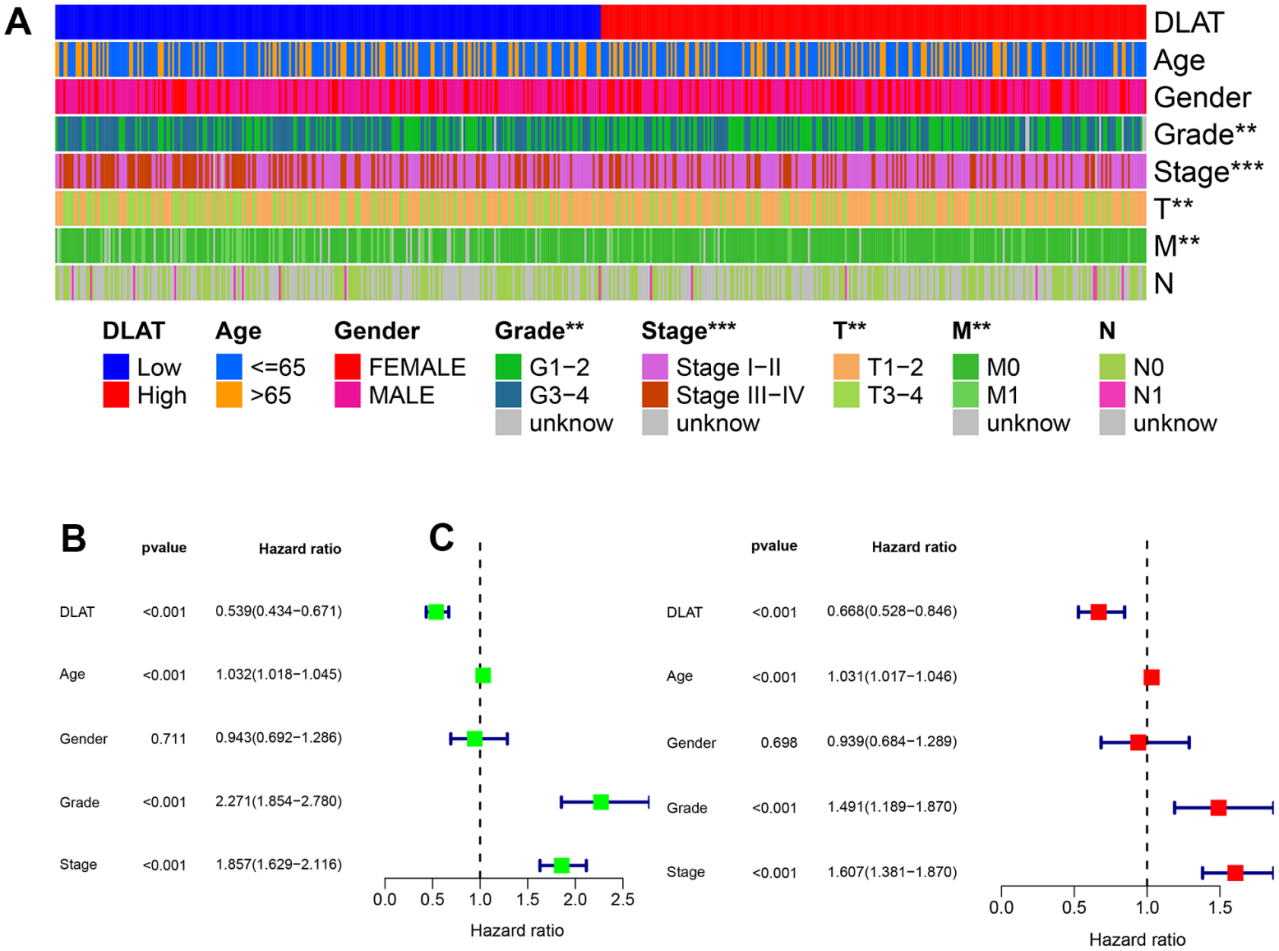
**Clinical correlation and independent prognostic analysis.** (**A**) Association between *DLAT* and traditional clinical features from TCGA. Blue on the left represents low *DLAT* expression, while red on the right represents high *DLAT* expression; and each line represents one clinical feature. (**B**, **C**) Univariate and multivariate Cox regression analyses confirmed the independent prognosis value of DLAT. *: *P*< 0.05; **: *P*< 0.01; ***: *P*< 0.001.

Clinical characteristics were compared between the *DLAT* high- and low-expression groups in the TCGA cohort. The results showed that *DLAT* expression was lower in grade 3–4 than in grade 1–2 tumors, in stage III–IV than in stage I–II, in T3–4 than in T1–2, and in M1 than in M0 metastatic stage (*P*< .01, Figure 3A). Univariate and multivariate Cox analyses showed that *DLAT* expression could be used as an independent prognostic indicator of OS in ccRCC, apart from other clinical factors (Figure 3B, 3C).

### *DLAT* correlation and functional analysis

**Figure 4:**
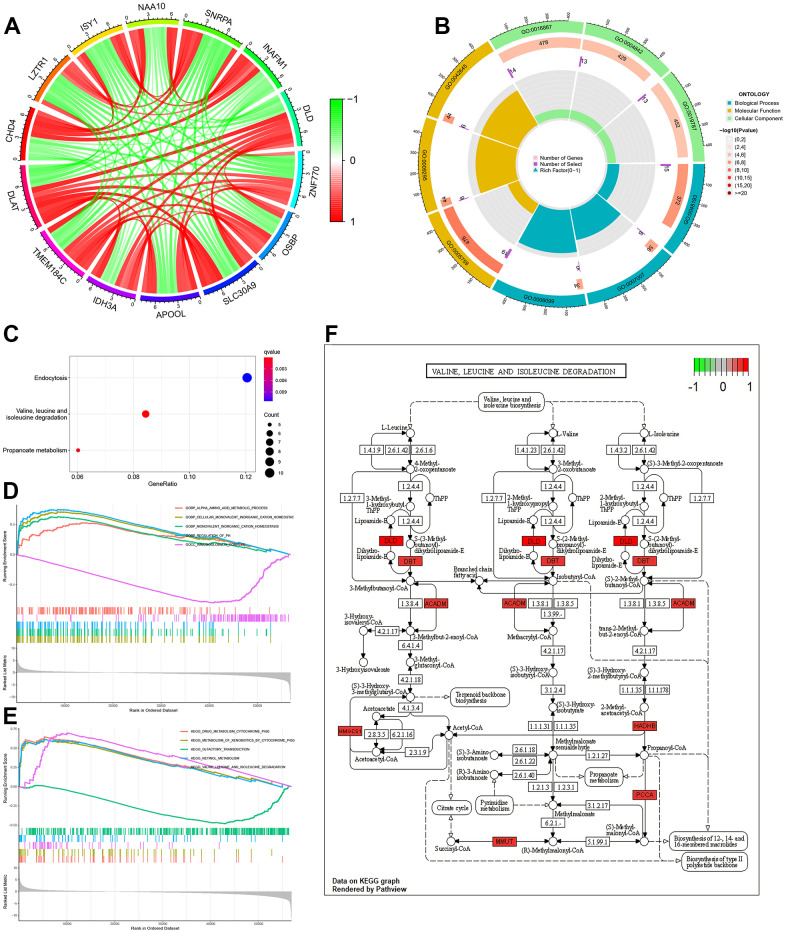
**Function, pathway and GSEA analyses of *DLAT*-related genes.** (**A**) Co-expression cycle graph of the 13 genes closely related to *DLAT*. The red line represents a positive correlation, while the green line represents a negative correlation. (**B**) GO circle graph of co-expressed genes. First circle: The top 9 GO terms, with the coordinate scale showing the gene number outside the circle. Second circle: The number of GO terms and Q values in the background gene. Third circle: Number of GO term associated genes. Fourth Circle: Abundance factor values for each GO term associated gene. (**C**) Bubble map of co-expressed genes from KEGG. (**D**) Function enrichment analysis between high- and low-expression groups of *DLAT* by GSEA. (**E**) Pathways analysis between high- and low-expression groups of *DLAT* by GSEA. (**F**) Pathways for valine, leucine and isoleucine degradation. Red represents key genes with high expression, while green represents key genes with low expression. BP: biological process; CC: cell component; MF: molecular function; GSEA: Gene Set Enrichment Analysis.

A total of 182 genes were related to *DLAT* with a correlation coefficient greater than 0.7. These included the copper cell death genes *ATP7A*, *SLC31A1*, *DLD*, and *DBT* (details are provided in Supplementary Material 1). The co-expression circular plot showed a correlation between 13 genes with the largest absolute values of the correlation coefficient, including *DLD* (Figure 4A). Biological processes (BP), cellular components (CC), and molecular functions (MF) were analyzed by GO enrichment analysis (Supplementary Material 2 and Figure 4B). KEGG pathway analysis showed that the co-expressed genes were mainly related to valine, leucine, and isoleucine degradation (Figure 4C and Supplementary Material 3).

GSEA was performed using the c5.go.v7.4. symbols.gmt dataset, and the results showed that the main function related to differentiate *DLAT* expression included alpha amino acid metabolic process, cellular monovalent inorganic cation homeostasis, monovalent inorganic cation homeostasis, regulation of PH and immunoglobulin complex (Supplementary Material 4 and Figure 4D). In addition, the main pathways related to *DLAT* included drug metabolism-cytochrome P450, metabolism of xenobiotics by cytochrome P450, olfactory transduction, retinol metabolism and valine, leucine, and isoleucine degradation (Figure 4E and Supplementary Material 5). The results of KEGG pathway analysis (Figure 4C) and GSEA (Figure 4E) showed that *DLAT* was closely related to the degradation pathways of valine, leucine and isoleucine. Further analysis revealed that the *DLAT*-related genes, including *DLD, DBT, ACADM, HMGCS1, HADHB*, *PCCA* and *MMUT* were functioned in this pathway (Figure 4F).

### Validation *in vivo* and *in vitro*

**Figure 5:**
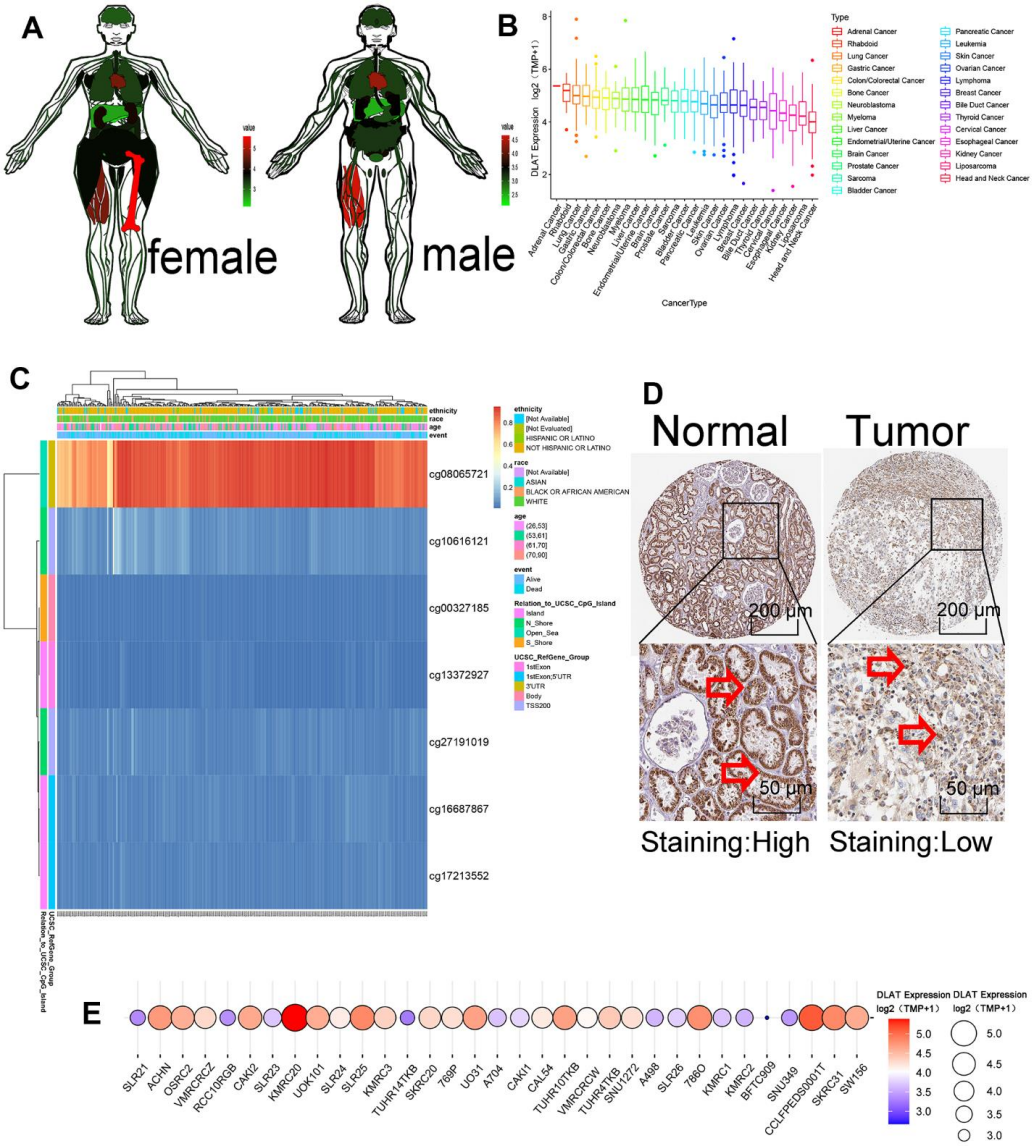
**Methylation and protein expressions of DLAT.** (**A**) *DLAT* expression levels in various human tissues. Red represents high expression and green represents low expression. (**B**) The expression level of *DLAT* in each tumor cell line. Decreased expression from left to right. (**C**) DNA methylation hot diagram of *DLAT*. Red represents high expression and blue means low expression. (**D**) *DLAT* protein level based on Human Protein Atlas. Normal tissues are on the left, while tumor tissues are on the right. The arrow is marked with DLAT protein. (**E**) *DLAT* expression at 33 kidney cancer cell lines. Red represents high expression, and blue represents low expression. The size of the circle represents *DLAT* expression.

In normal human samples in the GTEx database, *DLAT* was relatively highly expressed in bone, muscle and heart tissues and intermediately expressed in normal kidney tissue (Figure 5A). In the cancer cell lines in the CCLE database, *DLAT* expression was relatively high in adrenal cancer, malignant rhabdoid tumor, and lung cancer and relatively low in kidney cancer, liposarcoma, and head and neck cancer (Figure 5B). During mRNA transcription, various modifications such as methylation affect transcription results. In this study, we examined the prognostic value of *DLAT* methylation in KIRC using the MethSurv. The DNA methylation heat map showed the highest *DLAT* methylation level in cg08065721 and the lowest methylation level in cg11372927 (Figure 5C). The final protein expression level of *DLAT* was consistent with its mRNA expression level. *DLAT* expression was high in the tubules of normal kidney tissues and low in those of tumor tissues (Figure 5D). Among the 33 CCLE RCC cell lines, *DLAT* had the highest expression in the KMRC20 cell line and the lowest expression in the BFTC909 cell line (Figure 5E).

**Figure 6:**
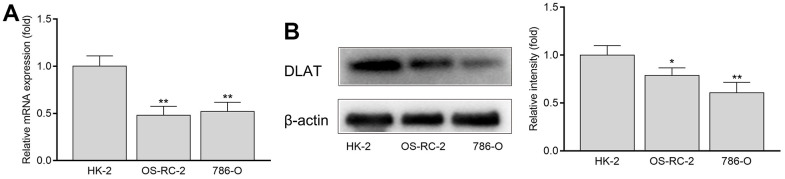
**The mRNA and protein expression of DLAT in HK-2, OS-RC-2 and 786-O cells.** (**A**) *DLAT* mRNA expression. (**B**) DLAT protein expression.

*In vitro* experiments showed that the expression of DLAT in normal renal cells (HK-2) was significantly higher than that in renal cancer cells (OS-RC-2 and 786-O), regardless of the mRNA or protein expression (Figures 6A, 6B).

### Immune infiltration and TME analysis

**Figure 7:**
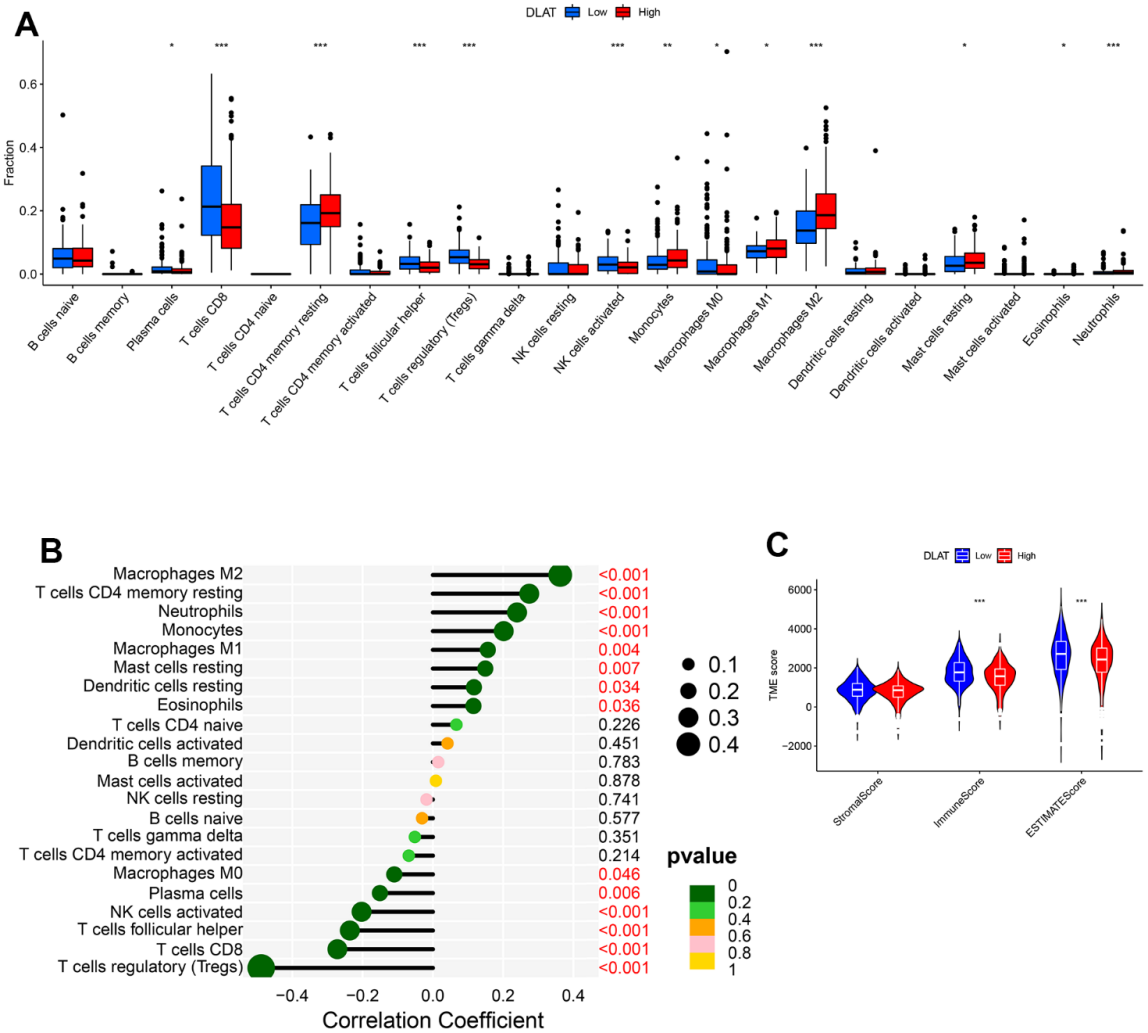
**DLAT-mediated immune invasion and micro-environment.** (**A**) Boxplot shows the differences of the 22 types of immune cells in high- and low-expression groups of DLAT. (**B**) Lollipop plot indicates the correlation between *DLAT* and 22 types of immune cells. (**C**) Violin graph shows the relationship between *DLAT* expression and TME (higher Immune and ESTIMATE Scores were witnessed in low-expression group of *DLAT*.).

*DLAT* expression is correlated to tumor immune infiltration and TME. We used the CIBERSORT method to explore 22 types of immune cells, 13 of which were found to be differentially expressed in ccRCC (*P*< .05, Figure 7A). Further analyses showed that *DLAT* was correlated with 14 types of immune cells (Figure 7B). Analysis of the TME showed that both ImmuneScore and ESTIMATEScore increased in the low *DLAT* expression group (*P*< .001, Figure 7C).

### Sensitivity to immunotherapy

**Figure 8:**
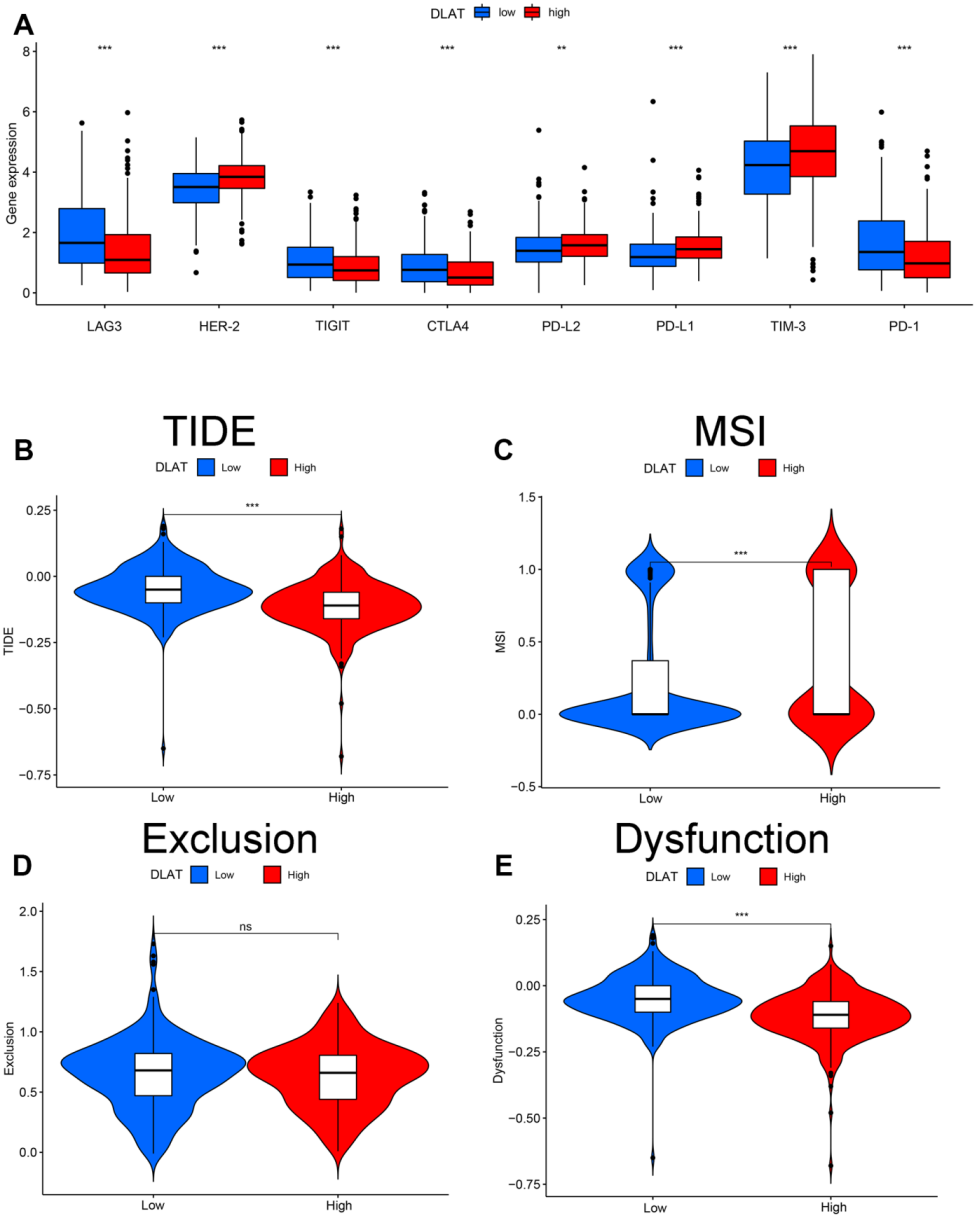
**Effect of *DLAT* on immune regulatory and TIDE in ccRCC.** (**A**) Gene expression of each immune checkpoint in high- and low-expression of *DLAT* from TCGA. *DLAT* expression distribution in the TIDE data set: (**B**) TIDE, (**C**) MSI, (**D**) Exclusion, and (**E**) Dysfunction.

Immune checkpoints are reliable molecules for assessing patient response to immunotherapy. Here, we assessed the differences in *DLAT* expression in relation to common immune checkpoints. The results showed that the expression of LAG-3, TIGIT, CTLA4, and PD-1 was upregulated, whereas that of HER-2, PD-L2, PD-L1, and TIM-3 was downregulated in the *DLAT* low-expression group (Figure 8A). In addition, TIDE-based simulations of the two main mechanisms of tumor immune evasion provide predictive outcomes for immunotherapy. Patients with *DLAT* low-expression showed a higher TIDE score and more obvious immune dysfunction and exclusion (Figures 8B–8E).

## DISCUSSION

In this study, we determined the role of *DLAT* in ccRCC patients, and discussed its main functions, especially its key role in immunotherapy.

*DLAT* is a component of the pyruvate dehydrogenase complex and participates in the TCA cycle in the glucose metabolism pathway. Lipid acylation of *DLAT*, which is induced by *FDX1*, directly binds to copper ions and contributes to form protein oligomers. Subsequently, it induces protein toxicity stress and promotes copper death, which enables a crucial role of *DLAT* during the process of cuproptosis. Therefore, *DLAT* has the potential to be therapeutic targets as well as the tumor prognostic indicators of ccRCC.

Multiple researches suggested that *DLAT* plays vital roles in various tumors. Through examination of multiple databases (TCGA, ICGC and GEO database), we confirmed that the expression level of *DLAT* in ccRCC was lower than that in normal tissues. Furthermore, 75 pairs of ccRCC and adjacent tissues in our database (NBUNH), as well as two renal cancer cells (OS-RC-2 and 786-O) and HK-2 normal cells were also verified, and similar results were found in the study of Jiang et al., whose research conducted a qRT-PCR study on 40 matched ccRCC tissues [16]. To further verify the function of *DLAT* in renal cancer cell lines, researchers constructed a lentivirus overexpressing *DLAT* vector that effectively inhibited the growth and metastasis of renal cancer cells [16]. Our results, based on the TCGA and ArrayExpress databases, showed that patients with high *DLAT* expression had better OS, which indicated that DLAT functions as a tumor suppressor (*P*< .05). According to ROC curve analysis, *DLAT* expression is a good indicator to distinguish benign from malignant renal tumors, which is consistent with the previous results reported by the study of Jiang et al. [16].

ccRCC has a unique metabolic profile, characterized by elevated levels of lactate, glutamate, pyruvate, glutamine, and creatine and decreased levels of acetate, malate, and amino acids such as valine, alanine, and aspartate [17, 18]. More severe metabolic dysregulation, including changes in valine, leucine, and isoleucine levels, has been observed in stage IV ccRCC [19]. Our results of KEGG and GSEA pathway analysis showed that *DLAT* was closely associated with the degradation pathway of valine, leucine, and isoleucine. Based on the above observation, we further examined the related genes in valine, leucine, and isoleucine degradation pathway. In this pathway, 7 genes (*DLD, DBT, ACADM, HMGCS1, HADHB, PCCA and MMUT*) were positively correlated with *DLAT* gene. Among these 7 genes, *DLD* and *DBT* were cuproptosis-related genes. Hence, we consider that the valine, leucine, and isoleucine degradation pathway is closely related to cuproptosis, of which *DLAT* may promote the progression of this pathway.

Consistent with our research, many studies have identified that high copper levels in tumors can regulate kinase activity, inhibit autophagy, and regulate fat metabolism [20–22]. Zheng reported that disulfiram/copper co-delivery triggered tumor cell autophagy, induced immunogenic cell death, activated tumor-infiltrating macrophages and dendritic cells, and primed T and NK (natural killer) cells, resulting in anti-tumor immunity and tumor regression [23]. We hypothesize that *DLAT* might promote apoptosis by influencing energy production. A recent research published in Science points out that cuproptosis is an unconventional mechanism of cell death concerning the protein lipoylation in TCA cycle [24]. Moreover, it is feasible to manage intracellular copper levels within a certain range to selectively kill tumor cells [25]. This is promising for novel insights to exploit copper toxicity in tumor therapy regimens.

Immunotherapy for tumors focuses on restoring host anti-tumor immune responses by blocking immune checkpoints [26, 27]. Huang et al. [28] reported that *DLAT* can promote the metabolism and biogenesis of liver hepatocellular carcinoma cells through MET kinase mediated phosphorylation. Fang et al. reported that *DLAT* was correlated with progression, chemo-resistance, and immune infiltration in pancreatic adeno-carcinoma [29]. It is reported that inhibitors of CTLA-4, LAG-3, PD-1, PD-L1 and other immune checkpoints have been shown to improve the OS of patients with advanced ccRCC [15, 30, 31]. The present study showed that immune checkpoints of LAG-3, TIGIT, CTLA4, and PD-1 expressions, as well as ImmuneScore and ESTIMATEScore were upregulated in *DLAT* low-expressed group. In addition, TIDE was applied to model the two major mechanisms of tumor immune evasion to provide predictive outcomes for immunotherapy. Results showed that the patients with low *DLAT* expression had higher TIDE scores compared with *DLAT* high-expressed group. Therefore, we consider that patients with low *DLAT* expression would develop more pronounced immune dysfunction and exclusion, resulting in poorer response to immunotherapy.

This study provides evidences for comprehensively understanding the role of *DLAT* on ccRCC. Although the robustness of our results was confirmed through multiple validations, such as datasets from multiple external databases, protein expression levels, and basic experiments, this study has some limitations. Specifically, the 76 ccRCC specimens collected were all early-stage tumors and did not cover all clinical statuses; therefore, additional immunotherapy cohorts are needed to validate and optimize the findings.

## CONCLUSIONS

We demonstrated the abnormal downregulation of *DLAT* mRNA and protein expression levels in ccRCC tissues. *DLAT* could serve as a biomarker to predict the clinical efficacy and sensitivity of targeted therapy in patients with ccRCC.

## MATERIALS AND METHODS

### Data collection and experiment queuing

RNA sequencing and clinical information of patients with ccRCC were obtained from The Cancer Genome Atlas Kidney Renal Clear Cell Carcinoma (TCGA-KIRC) database (https://portal.gdc.cancer.gov/; normal samples [N]=72; kidney tumor samples [T]=532). The International Cancer Genome Consortium (ICGC) (Renal Cell Cancer [RECA]-EU; http://dcc.icgc.org; N=45; T=91), ArrayExpress (E-MTAB-1980; https://www.ebi.ac.uk/arrayexpress/; T=101), and Gene Expression Omnibus (GEO; GSE36895; https://www.ncbi.nlm.nih.gov/geo/; N=29; T=23) databases were used for validation. The clinical data for each database are presented in Table 1.

**Table 1:** Summary clinical characteristics of ccRCC patients.

**Characteristics**	**TCGAN=532**	**ICGC N=91**	**E-MTAB-1990 N=101**	**GSE36895 N=29**
Age category				
<65/≥65/NA	333/199	57/34	52/49	15/13/1
Gender				
Male/ Female	345/187	52/39	77/24	17/12
Vital status				
Alive/ Dead	357/175	61/30	78/23	NA
Grade				
G1/G2/G3/G4/NA	14/228/206/76/8	NA	13/59/22/5/2	2/13/6/8/0
Tumor stage				
I/ II/ III/ IV/ NA	266/57/123/83/3	NA	66/10/13/12/0	5/2/3/6/13
T stage				
T1/ T2/ T3/ T4/ NA	272/69/180/11	NA	68/11/21/1/0	14/4/8/3/0
M stage				
M0/ M1/ MX	421/79/32	NA	89/12/0	16/4/9
N stage				
N0/ N1/N2 / NA	240/16/0/276	NA	94/3/4/0	11/3/15/0

NA: Clinical data are unknown.

Renal cancer samples from 75 pairs of ccRCC patients were collected from NBUNH. This study was approved by the Ethics Committee of Ningbo Urology and Nephrology Hospital. Written informed consent to participate in the study was obtained from all participants.

### Cell cultures

The normal kidney cell line (HK-2) and renal cancer cell lines (786-O and ACHN) were purchased from the Cell Bank of the Chinese Academy of Sciences (Shanghai, China). HK-2 cells were cultured in Dulbecco’s modified DMEM medium (HyClone Laboratories, Logan, UT, USA) and OS-RC-2 and 786-O cells were cultured in RMPI-1640 medium (HyClone, Logan, UT, USA). All cells were incubated at 37° C in 5% CO_2_ after supplementing the culture medium with 10% heat-inactivated fetal bovine serum (HyClone, Auckland, New Zealand), 100 U/mL streptomycin, and 100 mg/mL penicillin (HyClone, Logan, UT, USA).

### RNA extraction, reverse transcription, and qRT-PCR

Total RNA was extracted from clinical samples or cells using the E.Z.N.A.® Total RNA Kit (Omega Bio-Tek, Norcross, GA, USA). RNA (1 μg) was reverse-transcribed into cDNA using ABScript II RT Master Mix (ABclonal, Woburn, MA, USA). RT-qPCR was performed on a 7500 real-time PCR system with 2X Universal SYBR Green Fast qPCR Mix (ABclonal, Woburn, MA, USA) according to the manufacturer’s instructions. The primer sequences used were as follows: *DLAT*, sense: 5'-CCTCCCACAGGTCCTGGAAT-3',’ anti-sense: 5'-GTGCAATAACCCGACGAATGT-3'; *GAPDH*, sense: 5'-GGAAGCTTGTCATCAATGGAAATC-3’, anti-sense: 5'-TGATGACCCTTTTGGCTCCC-3’. Relative gene expression was normalized to that of GAPDH and the 2^− ΔΔCt^ method was used to calculate the relative expression levels of *DLAT*.

### Western blot analysis

Cell lysates were harvested in radioimmunoprecipitation assay (RIPA) buffer (Solarbio, Beijing, China) containing 1% PMSF protease inhibitor (Solarbio). Total protein concentration was calculated using a BCA Protein Assay Kit (Beyotime, Beijing, China). Total protein (20 μg) samples were loaded and separated using 12% SDS-PAGE, transferred to PVDF membranes, blocked with 5% non-fat dry milk, and incubated overnight with diluted primary antibodies against DLAT (Proteintech, Wuhan, China) or β-actin (Proteintech) at 4° C. The blots were then washed with TBST, incubated with horseradish peroxidase-labeled secondary antibodies (Boster, Wuhan, China), and visualized using an enhanced chemiluminescence reagent.

### Comparison of expression levels, Kaplan–Meier survival, and receiver operating characteristic (ROC) analysis

TIMER2.0 (http://timer.cistrome.org) was used to determine *DLAT* expression levels in various tumor types. Boxplots were used to visualize the expression levels of *DLAT* in discrete and paired tissues. Survival curves were generated to analyze the different survival outcomes in the high- and low-expression groups. ROC curve was drawn, and the area under the curve (AUC) was calculated to evaluate the specificity and sensitivity of *DLAT* expression in predicting benign and malignant tumors.

### Clinical relevance and independence analysis

The R package Complex Heatmap was used to draw heat maps to compare clinic-pathological parameters in the high- and low-expression groups. Univariate and multivariate Cox regression analyses were performed in R to assess the independence value of *DLAT* expression in distinguishing ccRCC in addition to traditional clinical characteristics.

### Functional and pathway analysis of co-expressed genes

|Pearson’s correlation coefficient|>0.7 and *P*< .001 were considered as the cutoff criteria for genes correlated to *DLAT*. The R packages circlize, corrplot, clusterProfiler, org.Hs.eg.db, dplyr, enrichplot, ggplot2, RColorBrewer, ComplexHeatmap, R.utils, and Pathview were used for co-expressed results and Gene Ontology (GO) and Kyoto Encyclopedia of Genes and Genomes (KEGG) analyses. The Molecular Signatures Database (MSigDB) gene sets c5.go.v7.4. symbols.gmt, and c2.cp.kegg.v7.4. symbols.gmt (http://software.broadinstitute.org/gsea/msigdb/index.jsp) were used for Gene Set Enrichment Analysis (GSEA).

### mRNA expression, methylation, and protein expression of *DLAT*

The Genotype-Tissue Expression (GTEx) database (https://gtexportal.org/home/) was used to verify *DLAT* expression in normal human organs. The Cancer Cell Line Encyclopedia (CCLE) database (https://sites.broadinstitute.org/ccle) was used to verify the expression levels of *DLAT* in tumor cell lines. The MethSurv tool (https://biit.cs.ut.ee/methsurv/) was used to evaluate the prognostic value of *DLAT* for different CpG methylation patterns in ccRCC patients. The Human Protein Atlas database (https://www.proteinatlas.org/) was used to determine protein expression levels of *DLAT*.

### Immune infiltration and tumor microenvironment (TME)

The CIBERSORT algorithm was used to evaluate the relationship between *DLAT* expression and the 22 types of immune cells. The lollipop plot allowed the visualization of the correlation between *DLAT* expression and immune cell types. The ESTIMATE algorithm was used to evaluate the immune microenvironment (ImmuneScore, StromalScore, ESTIMATEScore and TumorPurity) levels between high and low *DLAT* expression groups. These operations were performed in R using the packages limma, estimate, e1071, reshape2, vioplot, ggExtra, and ggpubr.

### Immune checkpoints and immune response prediction

The relationship between *DLAT* expression and hotspot immune checkpoints was visualized using box plots. The Tumor Immune Dysfunction and Exclusion (TIDE) database (http://tide.dfci.harvard.edu) was used to predict immunotherapy responses to differential expression levels of *DLAT*.

### Statistical analysis

All our operations and statistical analyses were performed using R software version v4.1.1 (https://www.r-project.org/). Statistically significant differences between two groups were determined using the Student’s t-test, and those among three or more groups were calculated using ANOVA. Pearson’s correlation was used to analyze the correlation between two different genes. A *P* value < .05 was considered statistically significant. NS, *P*> .05; *, *P*< .05; **, *P*< .01; ***, *P*< .001.

### Data availability statement

The datasets for this study can be found in ICGC at http://dcc.icgc.org, KIRC at https://portal.gdc.cancer.gov/, ArrayExpress at https://www.ebi.ac.uk/arrayexpress/, and GEO at https://www.ncbi.nlm.nih.gov/geo/. The authors confirm that the data supporting the findings of this study are available within the article and its Supplementary Materials.

### Consent

The consent was obtained from the patients for the publication of this paper.

## Supplementary Material

Supplementary Material 1

Supplementary Material 2

Supplementary Material 3

Supplementary Material 4

Supplementary Material 5
